# QueerVIEW: Protocol for a Technology-Mediated Qualitative Photo Elicitation Study With Sexual and Gender Minority Youth in Ontario, Canada

**DOI:** 10.2196/20547

**Published:** 2020-11-05

**Authors:** Shelley L Craig, Andrew D Eaton, Rachael Pascoe, Egag Egag, Lauren B McInroy, Lin Fang, Ashley Austin, Michael P Dentato

**Affiliations:** 1 Factor-Inwentash Faculty of Social Work University of Toronto Toronto, ON Canada; 2 College of Social Work The Ohio State University Columbus, OH United States; 3 Ellen Whiteside McDonnell School of Social Work Barry University Miami Shores, FL United States; 4 School of Social Work Loyola University Chicago Chicago, IL United States

**Keywords:** lesbian, gay, bisexual, transgender, queer, youth, photo elicitation, photo voice, grounded theory, online research, Canada

## Abstract

**Background:**

The experiences of resilience and intersectionality in the lives of contemporary sexual and gender minority youth (SGMY) are important to explore. SGMY face unique experiences of discrimination in both online and offline environments, yet simultaneously build community and seek support in innovative ways. SGMY who identify as transgender, trans, or gender nonconforming and have experiences with child welfare, homelessness, or immigration have been particularly understudied. A qualitative exploration that leverages technology may derive new understanding of the negotiations of risk, resilience, and identity intersections that impact the well-being of vulnerable SGMY.

**Objective:**

The objectives of the QueerVIEW study were to (1) enhance understanding of SGMY identities, both online and offline, (2) identify experiences of intersectionality among culturally, regionally, and racially diverse SGMY in Ontario, Canada, (3) explore online and offline sources of resilience for SGMY, and (4) develop and apply a virtual photo elicitation methodological approach.

**Methods:**

This is the first study to pilot a completely virtual approach to a photo elicitation investigation with youth, including data collection, recruitment, interviewing, and analysis. Recruited through social media, SGMY completed a brief screening survey, submitted 10 to 15 digital photos, and then participated in an individual semistructured interview that focused on their photos and related life experiences. Online data collection methods were employed through encrypted online file transfer and secure online interviews. Data is being analyzed using a constructivist grounded theory approach, with six coders participating in structured online meetings that triangulated photo, video, and textual data.

**Results:**

Data collection with 30 participants has been completed and analyses are underway. SGMY expressed appreciation for the photo elicitation and online design of the study and many reported experiencing an emotional catharsis from participating in this process. It is anticipated that results will form a model of how participants work toward integrating their online and offline experiences and identities into developing a sense of themselves as resilient.

**Conclusions:**

This protocol presents an innovative, technology-enabled qualitative study that completely digitized a popular arts-based methodology—photo elicitation—that has potential utility for contemporary research with marginalized populations. The research design and triangulated analyses can generate more nuanced conceptualizations of SGMY identities and resilience than more traditional approaches. Considerations for conducting online research may be useful for other qualitative research.

**International Registered Report Identifier (IRRID):**

DERR1-10.2196/20547

## Introduction

### Sexual and Gender Minority Youth (SGMY)

SGMY face unique challenges that impact their sense of self. Their experiences of exclusion and discrimination are important to examine to better understand the unique psychosocial and mental health needs of SGMY and identify instances of resistance and resilience [1]. Minority stress, which includes the stigma of living with a sexual and/or gender minority (SGM) identity [2], impacts the daily lives of SGMY through chronic discrimination, such as microaggressions and name-calling [3], and other acute events, such as physical and sexual violence [4]. Minority stress manifests in higher rates of mental health disorders for SGMY than for their heterosexual or cisgender peers, which may include depression, anxiety, suicide attempts, and posttraumatic stress disorder [5]. Alongside mental health ramifications, SGMY may also be at risk of later developing physical health conditions in response to their minority stress, such as cardiovascular disease, asthma, diabetes, and other chronic conditions [6]. The prevalence of such physical and mental health conditions is considerably higher for those facing multiple and intersecting vulnerabilities [5,6]. Although extant research has quantified the challenges experienced by SGMY generally, as well as several subpopulations [2-7], the experiences of most SGMY cannot be understood in “identity silos” but rather through an exploration of the complexity of the intersections in their daily lives [7]. This qualitative research protocol focuses on the development and implementation of QueerVIEW, a technology-mediated photo elicitation study that examined the intersectionality and resilience of SGMY who also have particular marginalizing experiences. QueerVIEW was a project developed by the Canadian Regional Network of the International Partnership for Queer Youth Resilience (INQYR), an international partnership of researchers working to address the needs of SGMY and their use of information and communication technologies (ICTs) within diverse global contexts. QueerVIEW utilized virtual photo elicitation methods to explore the complex and intersecting identities of SGMY who identify as members of at least one of four priority populations: (1) trans and gender nonconforming youth, (2) youth who have experiences with homelessness, (3) youth with current or past involvement in the child welfare system, and (4) youth who are immigrants, refugees, or newcomers to Canada.

### Trans or Gender Nonconforming Youth

Trans is defined as having a different gender than the gender assigned at birth [8-11]. Gender nonconforming and gender diverse is an identity endorsed by people whose gender expression differs from societal expectations of masculinity or femininity [8]. Accurate numbers of trans and gender nonconforming (TGNC) and gender diverse youth remain unavailable at the population level in countries such as Canada and the United States, as national censuses have historically asked for current gender identity via binary options (instead of multiresponse) and have not asked for gender assigned at birth [9]. Available prevalence data are often based on convenience samples recruited by independent researchers [9]. According to reports in the United States, between 0.7% and 1.8% of youth between the ages of 13 and 17 identify as trans [10,11], while Canadian estimates report that approximately 0.6% of the general adult population identifies as trans [12]. A recent study of 6309 SGMY in the United States and Canada found that 14.6% (n=924) identified as trans and 23.9% (n=1506) identified as gender nonconforming [13].

### Challenges Faced by TGNC Youth

Youth identifying as TGNC face a particular set of challenges, including feelings of invisibility, hypervisibility, and hostility [14]. Compared with their cisgender (ie, identify with gender assigned at birth) counterparts in the general population, TGNC youth have a higher risk of mental health issues such as psychological distress, self-harm, depression, and suicide, while nonbinary youth are more likely to report self-harm, as reported in the Canadian Community Health Survey [15]. Relatedly, alcohol use and victimization experiences have been found to be higher for TGNC youth than for the general youth population [16]. TGNC youth also reported significantly poorer health outcomes and utilization of health care services than cisgender youth [17]. TGNC adults experienced higher odds of discrimination, depression, and suicide attempts compared with cisgender lesbian, gay, or bisexual individuals [18]. Similarly, depression and anxiety were reported as occurring with higher effect sizes for TGNC college students than for their cisgender lesbian and gay peers [19].

### Experiences of Homelessness

Approximately 40% of the 35,000 to 40,000 youth who experience homelessness or housing instability in Canada during an average year identify as SGMY [20]. The term “homelessness” can encompass a range of unstable housing situations, including unsheltered (or absolutely homeless), emergency sheltered (those in overnight shelters or shelters specific to family violence), provisionally accommodated (temporary or insecure housing tenure), and at risk of being homeless (precarious housing or financial situations that may lead to homelessness) [21]. The most frequently cited cause of homelessness among SGMY is identity-based family conflict [22]. Compared with non-SGMY experiencing homelessness, SGMY are more likely to engage in survival sex work when homeless, engage in unsafe sex with their sex work clients, and have higher numbers of sex work clients overall than their cisgender heterosexual peers [23]. TGNC populations appear to be particularly at risk, with a sample of youth (younger than 18 years) and adult trans men experiencing significant and comorbid violence as well as physical and mental health problems. Meanwhile, homeless trans women report higher rates of posttraumatic stress disorder than other homeless individuals [24].

### Engagement in Child Welfare

Research from the United States on the prevalence of SGMY engagement in the child welfare system estimates that 15% to 34% of the approximately 350,000 individuals with a history of foster care involvement identify as SGMY at the point of intake [25,26]. Estimates of SGMY among the approximately 63,000 Canadian children in the child welfare system are unavailable, with no child welfare surveys currently collecting data on SGM identity [27] and large-scale reports not reporting on SGMY intake incidence [28]. Systemic limitations on collecting population-based data for Canadian child welfare agencies have been noted elsewhere [29]. SGMY experience higher rates of adverse childhood events than their peers [30] and are at a greater risk of involvement in the child welfare system, often as a result of their SGM status, as they may experience intrafamilial abuse, conflict, and rejection due to their identities [31]. SGMY in foster care report feeling less satisfied with their foster care experience than non-SGMY, are at greater risk for homelessness, and experience more placement breakdowns, resulting in greater emotional distress [32]. Research on SGMY in the child welfare system is often limited by small numbers of consenting participants because youth have concerns about disclosing their identities [33]. While in foster care, SGMY are often the victims of physical abuse, bullying, and harassment from caregivers and other youth [34], resulting in increased rates of posttraumatic stress and other mental health issues [35].

### Immigrants, Refugees, and Newcomers

Approximately 21.9% of the Canadian population (37.59 million) was born outside of the country [36]. SGM refugees, immigrants, and newcomers face unique challenges and stressors as a result of their migration and SGM status, including conflict in one or across many of their identity facets or affiliations [37]. Newcomer SGMY, or SGM individuals with landed immigrant status in Canada [38], are recognized as a particularly vulnerable group by the Canadian Immigration and Refugee Protection Act [39]. The Canadian settlement process can vary depending on the applicant’s status and type of claim [40], contributing to a large range of experiences for newcomers. SGMY newcomers often have complex interactions with immigration, health care, and employment systems, and frequently have experienced homophobia and racism within their own communities and families, and related to social service provision [41]. Stressors include difficulties accessing health care and employment, and stress associated with refugee claimant hearings. These hearings often emphasize a claimant’s ability to disclose and demonstrate their SGM identities, which may be unnecessarily difficult [41]. SGMY newcomers—particularly those who identify as trans females—report experiencing significant discrimination and bullying both in school and in their homes [42]. Many SGM immigrants to Canada discuss feeling disconnected from both their home culture and their Canadian culture because of their SGM identity, although many report that their SGM identity either becomes more important to them than their cultural identity or is integrated to allow them to live authentically [43]. Newcomer or refugee SGMY are a particularly understudied population, thus necessitating a greater understanding of their experiences in the research literature.

### Theoretical Approach

This study’s key theoretical framework is intersectionality, which not only explores distinct social identities, including their construction and intersections related to power, privilege, and experiences of discrimination, but also serves as a dynamic lens for investigating minority stress and resilience [43,44]. Focusing on a single identity may obscure the significance of other meaningful identities [45]. Intersectionality suggests that categories of oppression (eg, race, ethnicity, gender identity, sexuality, disability, and/or poverty) interact with and complicate an individual’s unique context and experience, and may contribute to a compounded experience of marginalization [46]. Although emergent qualitative research has explored the interaction of SGM and racialized identities, there is a paucity of literature utilizing technology and arts-based innovative methods to understand intersectionality factors and the experiences of marginalized SGMY [7].

### Rationale for Photo Elicitation

Visual data—such as photographs, videos, art pieces, and diagrams—have received increased interest and use in qualitative research [47], including as a tool to deepen conversations between participants and interviewers. Photo elicitation is a visual data method in which photographs provided by participants that capture relevant concepts under investigation are inserted into, and become the focus of, the research interview [48]. Photo elicitation offers a creative alternative to verbal-only methods of qualitative interviewing that is particularly suited to exploring intersectionalities. Images may evoke deeper elements of human consciousness than words alone, resulting in an interview process that elicits more information and evokes different types of information [48]. The use of photos to guide discussion and stimulate memory [48] has been demonstrated to increase participant-led dialogue [49], and can result in newfound insights compared with studies using more traditional methods to explore the phenomenon of interest [50]. Photo elicitation often results in richer data through the facilitation of rapport building between participant and interviewer [51] and by encouraging participants to provide a richer understanding of their experiences—including emotions, feelings, and ideas—rather than relying on researchers to impose their own assumptions, frameworks, or perceptions [52]. Images can ultimately promote a deepened dialogue and potentially introduce new dimensions that the researcher did not previously consider for their study [53]. 

Photo elicitation has been proposed as a participant-driven research methodology that is particularly well-suited for research among the adolescent population in general [54], and SGMY in particular. It reduces power differentials between researcher and participant [55], creating a “comfortable space for discussion” [56], and involving participants in a way that does not limit responses. Such advantages may be especially important for data collection with marginalized groups, such as SGMY [57]. By actively taking and selecting relevant photos for the interview, participants maintain agency over their participation in the research process. As a method, photo elicitation has been described as being capable of “empowering and emancipating participants by making their experiences visible” [57] and centering participant voices [58]. Photo elicitation interviews (PEIs), similar to semistructured interviews without visual cues, provide the researcher with an interview guide centering on relevant areas of interest, as well as the flexibility to allow for unexpected topics to emerge [59].

The increasing availability of ICTs has resulted in new opportunities and enhancements to the research process through digital data collection [60]. Qualitative interviews conducted online have been demonstrated to overcome financial, geographic, and physical mobility barriers for participants [61]. Online reviews [61], focus groups [62], instant messaging [63], and qualitative analysis of public message boards [64] are all well-documented methods for digital data collection in qualitative research. For use with adolescents, online interviewing may be particularly effective, given this age group’s comfortability, familiarity, and proficiency with ICTs [65]. Online interviewing with adolescents has demonstrated greater rapport building than in-person interviews and genererated similar amounts of youth self-disclosure [66]. Despite the potential for qualitative virtual PEIs for research with youth, there are no studies that have utilized these methods to date.

The use of technology-enabled photo elicitation methods may be particularly relevant for SGMY, who frequently use such technologies to develop their identities, access resources, and engage in online SGM communities [67]. SGMY also use ICTs to foster their coping skills and resilience [44]. As a result of widespread use and availability of ICTs, which permit easy collection and sharing of visual information (eg, smartphone cameras), youth are constantly engaged in recording their lives and experiences through photographs and videos. Virtual photo elicitation may offer the opportunity to advance insight into the intersectionality of SGMY’s lived experiences through the use of participant-recorded real-world data. As such, the QueerVIEW study used PEIs to explore the identity and resilience of SGMY experiencing compounded marginalization or vulnerability within the context of their online and offline lives. 

The research aims of QueerVIEW were to (1) enhance understanding of SGMY identities, both online and offline, (2) better understand experiences of intersectionality among culturally, regionally, and racially diverse SGMY in Ontario, Canada, (3) explore SGMY’s online and offline sources and processes of resilience, and (4) develop and apply a virtual photo elicitation methodological approach.

## Methods

QueerVIEW utilized a constructivist grounded theory framework in a virtual photo elicitation study to explore the intersectional identity experiences of SGMY in Ontario, Canada. Constructivist grounded theory concerns the construction of events, processes, and outcomes in order to study inequality by moving between theorizing and data collection. Constructivist grounded theory applies a critical lens and locates the research process within social, historic, and environmental conditions [68]. This form of inquiry involves co-constructing meaning through the development of emerging questions through the interactive engagement of the researchers with participants by posing critical questions from the inception of the project through the final analysis. For instance, in the case of QueerVIEW, this involved determining a new theme discussed by the participants, and choosing to pursue new questions in future interviews about that subject matter. In this way, constructivist grounded theory results in a deeper level of theorizing and unveiling of new critiques when compared with other qualitative methods [68]. Photo elicitation may be an ideal modality for constructivist grounded theory, as this method of interviewing can dismantle power differentials between participants and researchers while engaging participants in conversations far beyond the limitations of the interview guide [56]. Institutional review board approval was received from the University of Toronto’s Health Science Research Ethics Board (Protocol #37041), which included a waiver of parental consent for participants younger than 18 years of age due to the possibility that participants’ parents were not aware of their SGMY identities and that knowledge could pose a risk to participants.

### Participant Recruitment and Sampling

Participant inclusion criteria for this study included the following: (1) aged between 14 and 29 years, (2) self-identifying as an SGM, (3) residing in Ontario, Canada, (4) able to capture and submit photos, (5) able to speak and understand English sufficiently to participate in the interview, and (6) able to participate in an online interview. To clarify criterion 2, participants were eligible if they self-identified as a gender minority (ie, not cisgender) and/or a sexual minority (ie, not heterosexual). These inclusion criteria were determined by the research coordinator and participants themselves using participants’ responses to the screening survey (for criteria 1, 2, and 3) and through interaction with participants for the photo submission and interview scheduling (for criteria 4, 5, and 6). The four groups identified above—TGNC individuals, those with experiences with homelessness, those engaged in the child welfare system, and immigrants, refugees, and newcomers to Canada—were explicitly mentioned as priority populations in the recruitment flyer and screening survey. The age range was purposeful, given that heightened awareness of identity issues often occurs during the developmental periods of adolescence and early adulthood [68,69].

QueerVIEW recruited SGMY through a purposive and venue-based sampling approach. Purposive sampling recruited participants with a flyer, which was shared through the INQYR Canadian Regional Network, and distributed through paid Facebook and Instagram advertisements and via Twitter. Venue-based sampling was conducted concurrently with agencies in Ontario that serve SGMY communities and/or specialize in practice with TGNC youth, as well as youth experiencing homelessness, youth involved in the child welfare system, and newcomers to Canada. Additionally, universities located in Ontario, SGM-specific student clubs, and religious centers were also contacted for distribution of the flyer.

The recruitment flyer (Multimedia Appendix 1) directed potential participants to a QueerVIEW project page on the INQYR website [70], which included additional details about the study, animated consent videos that described the study’s purpose and process in an accessible way, and a link to the screening survey [71]. Further, a live action video of the interviewers was posted on the website and distributed on social media to make participants aware of the interviewers and study, to support self-efficacy, and to reduce anxiety.

### Consent

Due to the age of the participants in this study, as well as to the multiple times of engagement, informed consent was collected twice: (1) prior to a screening survey hosted by Qualtrics’ online survey software [72], and (2) prior to the interview. In the screening survey, consent information was provided in writing as well as in a specific animated video that used the written form as a script. Participants had the option to either read or watch the informed consent information [73] and, through the use of skip logic, were required to acknowledge they heard or read, understood, and agreed to participation before progressing through the online screener survey. The consent information was scanned for eighth grade readability [74] to ensure understandability and accessibility for all participants. It has been found that consent videos encourage more participants to carefully consider the implications of participation and increase their knowledge of their rights as participants [75]. At the end of the interviews, participants were asked if they would be interested in having their photos publicly displayed in an online gallery. If interested, participants verbally consented, and indicated which photos they did and did not want to be displayed. A separate consent form (Multimedia Appendix 2) will be emailed to participants closer to the gallery launch date (projected for fall 2020).

### Data Collection

#### Stage 1: Online Screening Survey

A brief online screening survey asked potential participants for their age, gender identity, sexual orientation, preferred pronouns, Canadian city and province of residence, current housing situation, and contact information, along with questions about their membership in the four priority subgroups (eg, “have you ever experienced homelessness or any form of housing instability, like couch-surfing, living in a shelter/hotel, or street-involved?”).

#### Stage 2: Participant Selection

The research team monitored the screening survey and met regularly to select participants who initially identified as members of multiple priority subgroups for interviews. Participants who identified with two or more subgroups were initially invited to interviews in order of screener survey completion, with SGMY with at least one priority group identification invited next. Selected participants were contacted by a research assistant through email or text (depending on their stated preference) with an invitation to participate in the study, and this continued until theoretical saturation was achieved.

#### Stage 3: Photo Selection and Submission

Individuals who agreed to participate were provided with instructions for taking, selecting, and submitting between 10 and 15 photos before the interview (Multimedia Appendix 3). These instructions asked participants to take and/or gather photos that represent the following areas: (1) who you are—how you see yourself in your online and offline lives, (2) how others see you in your online and offline lives, (3) what makes it hard for you to be who you are and what challenges do you face when trying to be yourself, and (4) what helps you be who you are and what gives you strength in the face of challenges. These areas were constructed by the research team (comprised of researchers, students, and people from the priority populations) based on their previous intersectionality research and emerging SGMY experiences to directly link the photo submission process to the research aims identified above. Participants were then instructed to upload their photographs via WeTransfer [76], an encrypted computer-file transfer service, before their interview.

#### Stage 4: Online Interviews

Two graduate research assistants trained in qualitative interviewing for photo elicitation studies conducted 90- to 120-minute online interviews using Zoom video conferencing. The research team used a Zoom Pro account, which provided up to 24 hours of meeting time, administrative feature controls including recording and screen sharing, customized personal meeting IDs, and a waiting room function. All of these functions were used during the interviews. Virtual interviews were recorded using Zoom’s record feature and participants were requested to keep their device’s camera on during the interview to collect both audio and video data. If participants required an in-person interview, these were also recorded via Zoom, directing the laptop’s camera and microphone toward the interviewer and participant during the meeting. Interviewers toggled between speaker view on Zoom and screen sharing to display the photographs provided by participants.

At the beginning of the interview, participants were provided with a general overview of the study and support with any technology-related questions. A semistructured interview guide (Multimedia Appendix 4) was carefully designed to capture participants’ experiences of intersectionality. Questions intended to uncover the personal meaning of minority stress and resilience in light of participants’ intersectional social identities [45], such as perceptions of their online and offline lives and how they navigate adversity. During the interviews, SGMY were asked to describe their photos, and the photos were used as prompts whereby interviewers asked for participants to elaborate at times or clarify statements. Participants referred to the photos when describing themselves, their challenges, and their strengths. When needed, the interviewers gently redirected participants’ sharing back to the photos provided by asking more in-depth questions about their emotions, senses, and recollections of their experiences (eg, “When you look at this photograph, how did you feel when it was taken? How do you feel now?”).

### Data Analysis

Data were analyzed using a constructivist grounded theory approach using NVivo 12 software by QSR International [68,77]. Constructivist grounded theory situates the researcher as co-constructing experiences and meaning so that the researcher’s reactions, interpretations, and descriptions of the interviews are captured in the analytic process [77]. Six independent coders analyzed and integrated three types of data from each participant—10-15 photos, video recordings of Zoom interviews, and textual transcripts of Zoom interviews—by importing the data into a single NVivo case and assigning codes based on the photos (which were discussed as part of the interview), interview content, and participant behaviours and affect (eg, facial expression, tone, etc) during the interview [78,79]. This triangulated approach of analysts conducting side-by-side coding of participant photos, nonverbal input from videos, and textual transcripts is one created by the authors to analyze technology-mediated research [80]. This analysis involved organizing the three data sources into initial and focused codes (primarily gerunds) that were combined in a single NVivo file to derive visuals (eg, data maps) for axial coding of the in-depth experiences of participants.

Six independent coders were assigned photos, videos, and transcripts to code simultaneously, with the spoken words as well as vocal intonations and body language of participants being coded as nodes and memos in NVivo [81]. The coding team is comprised of graduate research assistants who are ethnically diverse and predominantly identify as SGMY. Line-by-line coding was used to generate codes from the descriptions of the participants about their identities, technology use, and resilience through their selected images, transcripts, and interviews. Data were triangulated to capture multiple processes occurring simultaneously, such as themes discerned from the content of participants’ sharing and process changes such as verbal tone, body language, or displays of affect (eg, crying or laughing), as well as participants’ interactions with their environments (such as awareness of their parents in the house, internet disruptions, or introductions of pets). Annotations were used to describe processes occurring in the research process (such as interview interruptions, or instances when transcript content and nonverbal communication were discordant). Line-by-line open coding of the 30 interviews has been completed, each fully coded twice by two independent coders. Axial coding is underway to confirm codes against emerging themes and develop a model that explains intersectional conceptions of resilience and identity. A data map has been created using Mindmeister software (MeisterLabs GmbH) and is being edited by the coding team. To enhance trustworthiness in independent coders, research assistants were trained to utilize grounded theory, employ the “constant comparison” method, and maintain an audit trail. Further, the principal investigator hosted monthly meetings to discuss coding progress [82].

### Presentation of Findings

Once analysis is complete, member checking will be conducted whereby participants will be invited to a virtual group meeting to discuss the analysis and how it converges and/or diverges with their individual participation. A draft of this analysis in the form of a written report and a visual will be distributed in advance of this meeting; for participants who cannot attend member checking in the virtual group, they will have the option of emailing a written response to the analysis. Once the results are finalized, study findings will be disseminated to relevant research and practice communities. The results will be presented at local, national, and international social work, qualitative research, and SGM-specific academic conferences and symposia, and submitted for publication in peer-reviewed journals. A summary of the results will be made available on the INQYR website, with infographics, diagrams, and descriptions shared on social media with links to the website. The results will also be detailed in funder reports.

As participants provided specific consent and interest in the research team publishing their photos, the QueerVIEW project will culminate in the creation of an online photo gallery. With participants’ consent, voiceovers or text descriptors will describe the images, with a future goal of turning the online gallery into an interactive game interface. Images will have blurred identifiable information, will not feature faces, and will be protected to the greatest extent possible (such as excluding the ability to save image files and barring access from online search engines, such as Google Images). Participants will have access to the online gallery to view their images. An online gallery reception will be hosted for all participants, research team, INQYR partnership members, and interested community members to attend.

## Results

Data collection has been completed with a total of 30 interviews. Major emerging themes center on the process of deliberate yet differential approaches to identity formation among SGMY using online and offline mediums. Early results are promising, with virtual photo elicitation serving as a useful tool to deepen the interview process and provide a glimpse into the lives of SGMY participants. Participants took extensive time to consider, select, and submit their photos. SGMY reported caring about their photo selection and the study process because they wanted to select the best photos to represent themselves and because of their potential vulnerability in sharing personal photos.

## Discussion

### Recruitment Process

The research team noted that the recruitment stage of this study took longer than anticipated at 7 months in length (September 2019 to April 2020), although this is comparable to offline photo-based research [83,84]. However, the COVID-19 global pandemic resulted in an increase in participants finalizing their photo selections and scheduling interviews. The recruitment process was adjusted and streamlined by the research team, with a gentle time limit conveyed to participants, in order to decrease the amount of time between recruitment and interview to facilitate the research process. Among the four highly marginalized priority populations identified above, participants who were immigrants, refugees, or newcomers to Canada and those with child welfare experiences were particularly challenging to recruit. Targeted recruitment strategies with specialized agencies were needed to increase participation in this regard. Future qualitative photo elicitation undertaken by the research team will likely focus on recruiting participants with one or two priority experiences.

### Interview Process

The online interview process was very successful, with the research team observing that rapport was built more rapidly online than during in-person interviews of a similar nature, which corroborates extant literature [83], perhaps as a result of familiarity with online platforms and the comfort with online sharing for SGMY [47]. Participants expressed a sense of emotional catharsis at interview completion, with many stating that they felt better after the interviews and many developing a deeper understanding of their experiences or patterns of behaviour, which aligns with extant photovoice and photo elicitation research [83-86]. Such expressions of catharsis will be explored in the analysis and reported in the context of existing literature on the photo elicitation method’s potential for therapeutic benefit.

### Limitations

Protocol limitations include the entry criteria of English comprehension and ability to participate in an online interview. While this study prioritized immigrants, refugees, and newcomers to Canada, the study team only had resources available for recruitment and data collection in English. Additional resources for translation services for recruitment, screening, photo submission, and interviewing could have mitigated this language barrier. As this study also prioritized SGMY with experiences of homelessness, access to a device (eg, smartphone) and a private space for the interview could have also been a barrier. Since this study recruited across the Canadian province of Ontario (a large land mass) during the COVID-19 pandemic, and with limited resources, travel for in-person interviews and the provision of devices were not possible. Partnering with community organizations in recruitment may have mitigated the access barrier to some extent, before the COVID-19 pandemic shuttered in-person services, as participants could potentially have used organizational resources to participate in the study.

### Conclusion

QueerVIEW is an innovative study that leverages technology and visual arts-based research in a virtual photo elicitation method that advances a creative investigation examining resilience among SGMY. This protocol describes the successful implementation of a completely virtual research study that integrates digital photography with online recruitment, PEIs, data collection, and analysis to deepen exploration of SGMY experiences. The application of this protocol has determined that (1) youth take extroaordinary care in selecting photos, which should be accommodated in recruitment strategies and study timelines, (2) online PEIs can result in increased engagement and sharing by SGMY participants, and (3) technology-enabled PEI studies can contribute to a sense of catharsis for youth participants. The target populations of this study have been chosen based on their experiences of resilience, and the methods offer a means to facilitate their empowerment by fully immersing the research team in their experiences. It is through these methods, which privilege SGMY participant voices about their experiences and accessibility, that the researchers will be able to produce results that portray a robust picture of SGMY intersectional experiences.

## Appendix

Multimedia Appendix 1 QueerVIEW flyer.

Multimedia Appendix 2 QueerVIEW online gallery consent form.

Multimedia Appendix 3 Photo instructions.

Multimedia Appendix 4 Interview questionnaire.

Multimedia Appendix 5 Peer review reports.

## Abbreviations

ICTs: information and communication technologies
INQYR: International Partnership for Queer Youth Resistance
PEI: photo elicitation interview
SGM: sexual and gender minority
SGMY: sexual and gender minority youth
TGNC: trans and gender nonconforming
