# Supplementary material for: QueerVIEW: Protocol for a Technology-Mediated Qualitative Photo Elicitation Study With Sexual and Gender Minority Youth in Ontario, Canada
Source: JMIR Res Protoc. 2020 Nov 5;9(11):e20547. doi: 10.2196/20547 (PMC7677025; doi:10.2196/20547)
Supplement: Multimedia Appendix 1 [file resprot_v9i11e20547_app1.pdf]

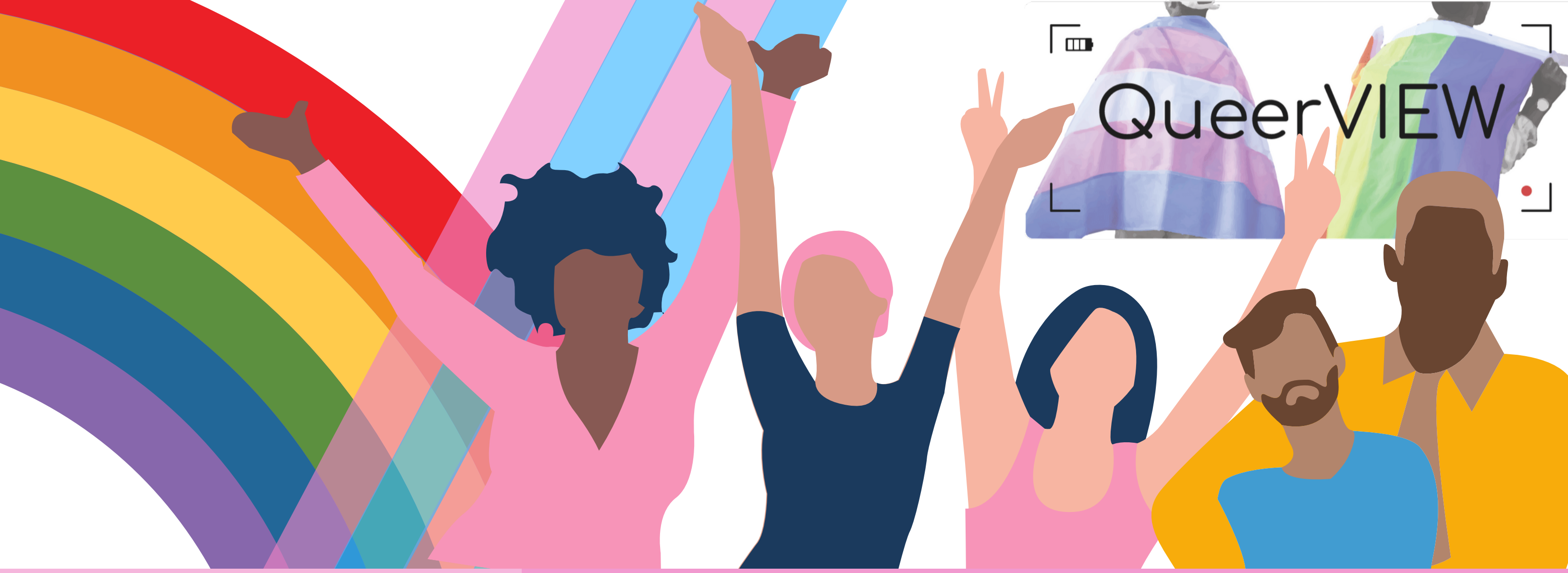

ARE YOU 14–29, IDENTIFY AS LGBTQ+, AND LIVE IN ONTARIO?

And do you identify with ONE OR MORE of the following?

- Identify as **trans, gender non-confirming, non-binary**
- Experience with **foster care** or child welfare
- Are a **newcomer** to Canada
- Experience with **homelessness** or couch-surfing

YOU DON'T NEED TO BE A PHOTOGRAPHER TO

SHARE YOUR STORY THROUGH PHOTOS

QueerVIEW is a study using photos of experiences of queer youth. It involves:

- A quick online **survey** to qualify
- Submit 10-15 **photos** from your phone/camera
- An **interview** (online or in Toronto)
- Receive a **$25 giftcard**

PARTICIPATE: visit our website at **www.inqyr.org/queerview**

CONTACT: **inqyr@utoronto.ca**

inqyr
queer youth & technology

FACTOR-INWENTASH FACULTY OF SOCIAL WORK
UNIVERSITY OF TORONTO
