# Supplementary material for: QueerVIEW: Protocol for a Technology-Mediated Qualitative Photo Elicitation Study With Sexual and Gender Minority Youth in Ontario, Canada
Source: JMIR Res Protoc. 2020 Nov 5;9(11):e20547. doi: 10.2196/20547 (PMC7677025; doi:10.2196/20547)
Supplement: Multimedia Appendix 2 [file resprot_v9i11e20547_app2.docx]

**QueerVIEW Online Gallery Consent Form**

Thank you for participating in QueerVIEW! [QueerVIEW](https://www.inqyr.org/queerview.html) was a photo-elicitation study for LGBTQ+ youth in Ontario hosted by the Factor-Inwentash Faculty of Social Work, University of Toronto. As part of QueerVIEW, you completed a brief screening survey, submitted 10-15 photos, and participated in an interview with either [interviewer names].

During the interview, your interviewer discussed how we are developing an online gallery for some photos to be featured. **From the photos you submitted, we would like to feature the selected images attached in the gallery. Would you be comfortable with these photos being featured in our online gallery?**As you’ll notice, we have removed any identifiable detail (such as by blurring faces), cleared photos of any data, and will post the images online in a way that they cannot be downloaded by others.

If you are comfortable with the photos being posted you may email me a brief description of the picture that we will post online alongside the image. Alternatively, we will use a quote from your interview (again, it will be completely de-identified) to describe the photo.

We would also like to include you as the creator of your work. **If you would like to be identified as an artist within the gallery and attributed to your work, please include your name to be published alongside your photos.** You are welcome to provide an artist statement (100-200 words), which is a brief description of yourself and your work to aid in the crowd’s understanding of your art.

Please reply to this email and let me know:

1. Would you be comfortable with the attached photos being published in our online gallery?

Yes                         No

1. Would you like to provide a new description of the image, or use your interview responses?

New Description                               Interview Responses

1. Would you like your name published in the online gallery alongside the images?

Yes                         No

- 1. If yes to #3, I would like my name published as: ________________
  2. If yes to #3, I would like to submit an artist statement (attached)

Thanks for your consideration, and for participating in the study!

[Research Coordinator Name & Contact Information]
