# Supplementary material for: QueerVIEW: Protocol for a Technology-Mediated Qualitative Photo Elicitation Study With Sexual and Gender Minority Youth in Ontario, Canada
Source: JMIR Res Protoc. 2020 Nov 5;9(11):e20547. doi: 10.2196/20547 (PMC7677025; doi:10.2196/20547)
Supplement: Multimedia Appendix 3 [file resprot_v9i11e20547_app3.docx]

**QueerVIEW Photo Instructions**

Thank you for your participation in the QueerVIEW study! One of the things that is most important to us in our research is your identity – groups and communities you feel you belong to (e.g., ethnic/racial group, peer group, cultural group, religious/spiritual group etc.), terms that you use to refer to yourself, parts of you that are influenced by these groups and other experiences. Even though this is a study about LGBTQ+ youth, we are interested in all your identities. So, we want to ask you to keep that in mind when you are taking your pictures.

Please take and/or gather 10-15 photos that represent different parts of you and your life that you will be willing to talk about with us during our interview within the next two weeks.

Specifically, please take and/or gather photos that represent the following areas:

1. Who are you - How you see yourself in your online life AND in your offline life
2. How others see you in your online life AND in your offline life
3. What makes it hard for you to be who you are? What challenges do you face when trying to be yourself?
4. What helps you be who you are? What gives you strength in the face of challenges?

We will be talking about each of the above topics during our interview, so please make sure you have at least one photo for each category.

**Note:** We welcome all kinds of photos with a couple important exceptions:

- NO photos of other people’s faces,
- NO sexually explicit photos

Before you attend an interview, we would like for you to submit your 10 to 15 photos on WeTransfer. WeTransfer is a secure cloud-based data sharing platform, that is used in this study to allow for the submission of photos for the interview.

1. Click (or enter into the address bar): [WeTransfer Link]
2. Click the (+) sign to add your photos, or if you have a folder dedicated to your selected folders, you can also click "Or select a folder" to upload the folder
3. In the "Email to" box, enter [Research Coordinator Email Address]
4. In the "Your Email" box, please enter the email that you used for the qualifying survey
5. In the "Message" box, please confirm your interest in participating in the interview

Once we receive your photos, we will contact you to confirm the interview date and location.

If you have any questions before your interview, please feel free to contact our research coordinator at [Research Coordinator Name & Email Address]

Thanks for participating!
