# Supplementary material for: QueerVIEW: Protocol for a Technology-Mediated Qualitative Photo Elicitation Study With Sexual and Gender Minority Youth in Ontario, Canada
Source: JMIR Res Protoc. 2020 Nov 5;9(11):e20547. doi: 10.2196/20547 (PMC7677025; doi:10.2196/20547)
Supplement: Multimedia Appendix 4 [file resprot_v9i11e20547_app4.docx]

**QueerVIEW Interview Guide**

[Interviewer signs onto Zoom and hits record.]

**Hi! I am [Name], an interviewer on this QueerVIEW study.**

Thank you for submitting your photos and participating in the interview! Our discussion is really about you. I will be asking you some questions about your photos to better understand your experiences and your point of view. You can skip any question that you do not want to answer.

One of the things that is most important to us in our research is your identity – groups and communities you feel you belong to (e.g., racial, cultural, religious or spiritual, etc), terms that you use to refer to yourself, parts of you that are influenced by these groups. Even though this is a study about LGBTQ+ youth, we are interested in all of your identities. So we want to ask you to keep that in mind when talking about your pictures.

Just a reminder that we are recording this so that we can analyze your interview but it is confidential. The recording will only be shared with our research team and housed in an encrypted and locked file. Do you have any questions?

Okay let’s get started! I am going to share the screen with your pictures so we both are looking at the same one. Which picture would you like to start with?

[Interviewer pulls up first photo]

- ***“Please tell me how this photo/these photos represent…” or “Talk to me about why you decided to take this photo to represent…”***
  - “who you are in your online life”
  - “who you are in your offline life”
  - “how others see you in your online life”
  - “how others see you in your offline life”
  - “the challenges you face in trying to be yourself”
  - “the things that help you”
- Potential probes for 1. a & b: ***“If you had to say what things make you ‘you’, what would these things be?”***

Potential probes for 1. a & b: “***Are there any parts of what make you ‘you’ that are sometimes in conflict with each other or that don’t always fit together well?” “What about parts that fit together well and feel good together?”***

- Potential probes for 2. a & b.: ***“If I asked a friend of yours from your offline life to describe you, what do you think they would say?” “If I asked a friend of yours from your online life to describe you, what do you think they would say?”***
- Potential probes for exploring relationships between 2 a. & b: ***“Talk to me about the similarities and differences between how you see yourself and how others see you? Does it change when you are online or offline? Why do you think that is?”***
- Potential probes for 3: ***“what were the times that you felt more resilient or strong?” “Can you tell me about a time that you felt particularly strong when facing a challenge? What was it about that time that made you feel particularly strong?”***
- Potential Probes for 4. ***“Were there times that you felt less resilient or strong?” “When you fall down, have a problem, or really struggle, how do you deal with it?” “How do you deal with difficult times and experiences?” “What has helped you to bounce back?”***
- Final probe: ***“Is there anything else that you think is important for me to hear or understand about you and your experience?”***

Thank you so very much for sharing your experience with me. I have learned so much from this conversation. I just have one more question.

In terms of next steps, we are going to analyze all of the interviews to try to better understand the collective experiences of queer youth. We are also going to develop an online gallery for some photos to be featured (if it makes sense to you). From the photos you submitted, would you be comfortable with some or all of them being featured in public presentations and our online gallery? You can always take more time or change your mind now or later. If so, I will ask you to also write a brief description of the picture in text or audio and send it to me so we can add it when the project completes.

**[If participant consents to sharing some photos, discuss which ones are okay to share].**

Okay, you will hear from us during the next day or so as we send your $25 Amazon gift card. Do you have any questions for me? If not, you can always email me later.

Thanks again for participating in the interview! Have a great day!

[Turn off Zoom and wait for the video to be ready to save. Save the video.]
