# Supplementary material for: QueerVIEW: Protocol for a Technology-Mediated Qualitative Photo Elicitation Study With Sexual and Gender Minority Youth in Ontario, Canada
Source: JMIR Res Protoc. 2020 Nov 5;9(11):e20547. doi: 10.2196/20547 (PMC7677025; doi:10.2196/20547)
Supplement: Multimedia Appendix 5 [file resprot_v9i11e20547_app5.pdf]

SSHRC CRSH

Expert Panel Report

Application number:	895-2018-1000
Institution:	University of Toronto
Project Director:	Shelley L. Craig
Project Title:	'Queery'ing resilience: Leveraging information & communication technologies to negotiate gender and sexual minority youth identity and wellbeing within diverse global contexts

Expert Panel evaluations are intended to provide the Selection committee with an in-depth analysis of the strengths and weaknesses of a proposal, so that it can determine the relative merit of a project compared to the cohort and make funding recommendations to SSHRC.

Expert Panel Agreement

- The information provided in the application is protected by Canada's *Privacy Act* and was made available to Expert Panel members for review purposes only. The Expert Panel has therefore agreed to treat all the material from the above-mentioned file as strictly confidential. They have agreed to ensure the destruction of the said materials once their duties have been completed.
- The Expert Panelists have attested that they are not in a conflict of interest with the project directors, or any co-applicants, collaborators and/or partner institutions.
- The Expert Panelists have agreed that the information presented in the following pages reflects the consensus reached in their deliberations on **January 18, 2018.**

Challenge—The aim and importance of the endeavour

Strengths

- The panel was impressed with this proposal, its scope, and the research focus (the impact of Information and Communication technologies [ICTs] on Gender and Sexual Minority Youth [GSMY] wellbeing). The project is poised to offer a novel contribution to our knowledge on several fronts: the resilience of Gender and Sexual Minority Youth, the role of Information and Communication Technologies in development and well-being, and current social practices of GSMY.
- The panel found the project to be particularly strong in terms of its theoretical framework which is informed by multiple approaches and disciplinary perspectives from psychology, social work, and public health, the appropriateness of the methods, the clarity of the mechanisms to provide training and mentoring to students and emerging scholars, and the potential for long-term viability. The project has high potential for impact in the social sciences and humanities across disciplines (sociology, psychology, public health, gender and sexuality studies, information technology and communication studies). Furthermore, the focus on sociocultural and geographic diversity elevates the impact significantly. Nonetheless, the panel identified a few weaknesses of the project's theoretical framework which are outlined below in "Weaknesses."
- The proposed project has many strengths in terms of its in-depth and specific focus on ICT use and engagements by GSMY in relation to local and contextually bound research and intervention, in conjunction with a transnational process of sharing and linking skills, information, and practical knowledge. The panel pointed out that very little research on queer youth has attended to the international flows and differences across national contexts. As such, the global reach, while challenging at the implementation level, is a unique element of this proposal.
- The panel positively noted the balanced use of both quantitative and qualitative methods, and the project's recognition of the importance of hybrid and situated approaches that emerge out of the social everyday worlds of young people.
- The panel pointed out that it is important that the focus on media is expansive, to consider a range of digital modalities including devices, social media platforms, games, and other interactive media. Considering that technologies change and proliferate, this project is flexible to take into account the heterogeneous, mobile and precarious ways in which ICTs are used by youth. The use of digital technologies to both study and also reach out to youth is significant and helps to connect to the life worlds of youth on/offline. The proposed technologies to help build resilience are also innovative in terms of situating youth as participatory actors within the process of their empowerment. This helps overcome the ways marginalized youth are passively framed as "victims" in previous research.
- Considering that so much research on youth and media is grounded and oriented in heteronormative ways, the panel found that this project is very strong in providing a comprehensive foundation for extensive and comparative work on marginalized youth.
- The project's intersectional focus is also valuable and attends to the diverse situations and experiences of youth in relation to cultural and social differences. Accounting for differences within as well as between regions will help to overcome the universalizing tendencies that often frame queer youth in static and homogenous ways. It is also vital in recognizing the ways sexuality and gender are interwoven with many other dimensions of identity and structural

Challenge—The aim and importance of the endeavour

conditions of inequality. However, the panel would have appreciated further precisions, which are described below in "Weaknesses".

- The panel also noted that most research to date is small scale and fragmented so that it is difficult to make connections between the results and build upon the work in a systematic and cumulative way. This large scale project would be the first of its kind and help to legitimize and foster the study of queer youth as an academic subject across disciplines.
- The panel remarked that all aspects of the project involve the training and participation of graduate students. This will encourage and help students who want to work on queer youth projects. It will offer institutional validation and support to queer youth oriented thesis work and postdocs, something that has been lacking, leaving students to work in isolation and without much funding if they chose to work on GSMY topics. This is incredibly important as many students struggle to work on those areas of youth experience that are socially invisible and subject to many dimensions of oppression and injustice. The panel sees this as being a very strong site from which to attract students working in this area and provide a collective and institutionally vibrant locus of collaborative learning.

Weaknesses

- While the proposal is largely focused on the logistics of facilitating coordinating regional networks and technologically driven research, the panel noted that an equal emphasis needs to be on accessing and sharing interdisciplinary bodies of knowledge of the pre-existing work that has a theoretical sophistication and reach. While the panel considers this fully possible within the terms of the research process proposed, it nevertheless finds that the theoretical foundations of the project are weak in terms of its lack of emphasis on cultural and media studies; given the focus of the proposal, the panel finds that the proposal will benefit from a more sustained and substantive attempt to incorporate the work by scholars beyond the disciplines of psychology and social work. Indeed, while the proposal makes references to a broad field of literature on queer youth, it is primarily centered on psychological and social work studies. Considering that some of the most interesting and exploratory work on queer youth and social media is situated within gender studies, education studies, cultural studies, critical youth studies as well as communication and media studies, the panel found that more attention needs to be paid to the emerging work in these areas that theorize the very complex relations and identifications of youth as they use and make media. In fact the most interesting qualitative work on queer youth offers dynamic conceptual tools that the panel would've liked to see this project draw upon.
- Furthermore, the panel found problematic some gaps. For example, although the author talks of positioning Canadian researchers as experts in the field of GSMY, many current Canadian experts in the field of GSMY were not cited. For example, Elizabeth Saewyc, at UBC directs a large interdisciplinary international research consortium, SARAVYC (Stigma and Resilience Among Vulnerable Youth Centre), with a big focus on GSMY—and this research output seems to have been omitted.
- The panel found that notions such as "at risk" youth and even the key term "resilience" would benefit from robust theorization.
- The panel would've expected more methodological diversity, especially given the expertise of some of the team (e.g. Carrillo). Indeed, the panel noted an over-reliance on self-report measures, and pointed out that the project would benefit from use of ethnographic or similar methods.

Challenge—The aim and importance of the endeavour
- The panel would have liked to see more clarification and details on what intersectionality entails. While national, regional and sociocultural differences are recognized throughout, the panel noted that questions of race/ethnicity lacked in specificity while questions of class and economic inequality were not raised. This is a weakness as the digital divide is a systemic problem and the "participatory gap" often leaves out the most marginalized youth so needs to be focused on in any work on ICTS and GSMY.   - The panel remarked that that the community organizations included as partners are all very established and adult centered. There are also many small organizations and online groups that are run and driven by queer youth and the panel found that their absence here is a weakness. Outreach and involvement of these youth initiatives would be very productive sites of engagement. The panel judges that this can be addressed within the terms of the project outlined.   - The panel also questioned the age boundaries of the research. It finds that 16 is quite old as a minimum age group and notes that there will be many queer youth left out of this process. Many youth in junior high and early high school articulate their gender and sexual identities and experience complex oppression as well as being actively engaged in online communities and microblogging. The panel therefore recommends that the project consider lowering the age to 12. This is fully possible within the framework proposed and would enable more youth to have a voice within the terms of its vision.   - In spite of an emphasis on a bilingual approach to the project, and in spite of Canada's bilingual reality, the panel remarked that French was left out of this project; no visible attempt has been made to include French-speaking GSMY scholars in either the Canadian context or in Europe, and it was not clear what efforts will be made, if any, to translate the Resilience Enhancement Products, for example, into French (even if there are no French Regional Networks). The panel thus recommended the incorporation of French into the project. It furthermore noted that this is fully possible within the terms of the project, as consideration is given to the question of language barriers and translation is factored into the budget.

Feasibility—The plan to achieve excellence
**Strengths**
- Overall, the panel was impressed with the quality and genuineness of the formal partnership, the expertise of the team, the appropriateness of the partner organization, the appropriateness of the requested budget, and the quality of the knowledge mobilization plans.   - The panel found the proposal to be very well organized and thorough in its planning at every level. A great deal of work and preparation has been done to establish partnerships with a broad range of academic and community organizations. There are established connections to these groups based on previous research projects and so they are grounded in established histories that have proven to be productive. The level of involvement of partner organizations and others is particularly impressive.   - The panel also found that the timeline is mapped out very clearly and logically. The objectives seem feasible within the proposed timeframe. Meetings are scheduled frequently to coordinate local and international partners. The phases are continuous and balanced and seem well paced so that the required work can be done within the proposed limits. The very elaborate

Feasibility—The plan to achieve excellence
governance procedures included in the application will help ensure the research unfolds in systematic ways according to the timeline.<br>• The academic co-applicants and partners are very experienced and include a range of disciplinary backgrounds and their proven research and publication output is outstanding and focused on gender and sexual minority subjects. It appears that linkages between the academic members have been established over many years and so this will benefit the future process. Also many within the academic team have worked on big projects and supervised graduate research training. They are all senior and experienced academics with a proven track record.<br>• The panel judged that a key strength is the breadth of community organizations that will participate. The balance and goal of knowledge exchange between academic and non-profit groups is strong.<br>• The panel commended the impressive level of commitment from the host institution and partner organizations in terms of cash and in-kind support. The project appears to be very strong in the promise by local and international partners to contribute resources.<br>• The panel also noted that the proposal lays out a strong, appropriate, and extensive knowledge mobilization plan and an appropriate dissemination strategy. It has incorporated innovative digital media projects and products that will help in the creation of accessible and diverse formats. Although subject to some limitations, the translation of materials is also welcomed by the panel.
**Weaknesses**
• The panel suggested that more scholars trained in cultural and media studies be included within the process since this a project that is centered on ICTs at every stage. While hiring technicians and staff with technological skills is necessary, the panel would have liked to see the participation of more experts in youth and digitial media. This can easily be achieved as there are many students and established scholars in this field.<br>• The panel noted that greater specificity about academic publishing outlets would’ve been beneficial. Few outlets are named, and it is not clear how wide their reach is within the social sciences and humanities.

Capability—The expertise to succeed
**Strengths**
• The panel judged that this project, under the direction of the project director, is highly likely to succeed.<br>• The panel remarked that the project director is positioned in an excellent way to carry out this project. She has an impressive record of research and publications and has undertaken many projects that are directly related to this work. Her dedication to this area is evident in terms of a long history of both academic research and writing along with community based work. She has focused directly on queer youth and digital technologies in other collaborative projects in which she was a leader. She has clearly proven her ability to foster networks of scholars working on queer youth. Similarly, the co-applicants also have very strong backgrounds and proven records as scholars in their fields. They seem equally qualified and since the regions will be chaired and

Capability—The expertise to succeed
governed by leading academics it is notable that they are very experienced and have all worked on large collaborative projects in the past. There is a consistency in the project since they have experience working in complex formal partnerships which will make this a strong collective. There is also evidence of the development of scholars through mentoring. Based on their CVs and the requirements of the project, the panel is convinced that there is a very strong promise of future contributions.
**Weaknesses**
• The panel did not find any weaknesses.
